# Access to infertility services in Canada for HIV-positive individuals and couples: a cross-sectional study

**DOI:** 10.1186/1742-4755-7-7

**Published:** 2010-05-12

**Authors:** Mark H Yudin, Heather M Shapiro, Mona R Loutfy

**Affiliations:** 1 The department of Obstetrics and Gynecology, St. Michael's Hospital, Toronto, Ontario, Canada; 2 The department of Obstetrics and Gynecology Mount Sinai Hospital, Toronto, Ontario, Canada; 3 The department of Internal Medicine, Women's College Hospital, University of Toronto, Toronto, Ontario, Canada

## Abstract

**Background:**

Family and pregnancy planning issues are important among human immunodeficiency virus (HIV)-positive individuals and couples. However, access to fertility services may be limited for this population. The objective of this study was to estimate the types of services available in fertility clinics in Canada for these individuals.

**Methods:**

A survey was sent to all registered fertility clinics in Canada to assess the availability of services (investigations and treatment) for infertility and/or viral transmission risk reduction in achieving pregnancy. The proportion and location of clinics willing to carry out investigations and treatments were determined. Logistic regression analysis was performed to assess differences in response rates, investigations, and treatments by province and by couple scenario.

**Results:**

Completed surveys were received from 23/28 (82%) of clinics across eight Canadian provinces. Seventy-eight per cent (18/23) were willing to accept HIV-positive individuals in consultation, and 52% had actually seen at least one HIV-positive man or woman in the previous year. Clinics in every province were willing to offer infertility investigations, but only clinics located in five provinces were willing to offer fertility treatments. The most commonly available treatment was intrauterine insemination for couples in which the female partner was HIV-positive (52%). Other techniques, such as sperm washing (26%) or in vitro fertilization (17%), were less commonly offered. A smaller number of clinics were willing to offer risk reduction techniques in achieving pregnancy.

**Conclusions:**

Access to infertility investigations and treatments in Canada is limited and regionally dependent.

**Trial Registration:**

Registered with ClinicalTrials.gov at http://www.clinicaltrials.gov, registration number NCT00782132.

## Background

Since the identification of the human immunodeficiency virus (HIV) and the acquired immunodeficiency syndrome (AIDS) in the 1980s, there have been significant advances made in the management and long-term prognosis for infected individuals. In the early days of the epidemic, most people diagnosed as HIV-positive were not expected to live a long lifespan. Currently, with the advent of combination antiretroviral therapy (cART), and the ability to reconstitute the immune system and keep viral loads to undetectable levels, HIV-positive individuals may live a healthy and productive life for years to decades after diagnosis. With this progress, family and pregnancy planning issues have become important for HIV-positive men and women.

Worldwide, it is estimated that 33.2 million people are infected with HIV. In Canada, women accounted for 27.8% of positive HIV tests in 2006 [1]. Among these women, at least 70% were of childbearing age [2]. Similarly, most HIV-positive men are fertile and have the potential to have children.

Living with HIV/AIDS may modify, but does not appear to remove the wish to have children. Several studies performed worldwide have documented that a substantial proportion of HIV-positive men and women have expressed the desire or intention to have children [3-5]. As life expectancy among patients infected with HIV has increased, there has been a need to allow this group of people to have access to advanced reproductive technologies and fertility treatments. Since the early years of the HIV epidemic, the treatment of fertility issues in couples in which one or both partners was HIV-positive has been controversial. However, more recently it has been argued that it is unethical to withhold fertility services to these individuals or couples [6-8]. Despite good evidence for the safety and efficacy of advanced reproductive technologies in HIV-infected couples [9-12], access to clinics providing these services may be limited. Barriers to access include not only ethical issues, but also technical difficulties related to the handling of specimens and training of staff. In both the United Kingdom and Australia, studies have been performed to evaluate the accessibility of fertility services for HIV-infected couples [13,14]. The results of these studies were disappointing, with limited access for both investigations and treatments.

The primary objective of this study was to determine the proportion and location of fertility clinics in Canada that would provide advanced reproductive technologies to HIV-infected individuals and couples. The secondary objectives were to determine how many clinics would provide specific services for infertility or for viral transmission risk reduction strategies to achieve pregnancy.

## Methods

Prior to initiation of the study, approval was obtained from the hospital Research Ethics Board. To assess available services, a questionnaire was sent to fertility clinics across Canada (Appendix A). This questionnaire was based on those used in the United Kingdom and Australia studies, as there was no other validated survey instrument available [13,14]. Prior to study initiation, study authors and other physicians reviewed versions of the questionnaire for face validity, and changes were incorporated for content validity and clarity. The final version was five pages long and contained 25 questions. Most questions were in yes/no format and assessed the availability of services (investigations and treatments) for HIV-positive men and women for infertility and/or viral transmission risk reduction in achieving pregnancy.

A list of all fertility clinics in Canada was compiled from the Canadian Fertility and Andrology Society. An introductory email was sent in November 2007 to the Medical or Laboratory Director of each clinic explaining the study and asking for participation. If the clinic agreed to participate, the survey was emailed. Respondents were permitted to return completed surveys by email or fax. Non-responders were re-contacted three times by email, and then by fax.

A sample size of convenience was used for this study as all clinics registered with the Canadian Fertility and Andrology Society were included. Completed questionnaires were sent to one of the authors (MY) and data was entered into an Excel spread sheet. The statistical analysis consisted mainly of summary statistics to report on the proportion and location of clinics willing to carry out infertility investigations and treatments for HIV-positive individuals and couples. Finally, we assessed how many clinics had specific policies relating to the investigations and/or treatments of HIV-positive individuals and couples. Using SAS 9.1, logistic regression analysis was performed to assess differences in response rates, investigations, and treatments by province and by couple scenario.

## Results

There were 28 clinics located across eight Canadian provinces. Completed surveys were received from 23/28 clinics, for a response rate of 82%. Table 1 presents the response rate by province. Using logistic regression analysis, there were no significant differences in response rates by province (p > .05).

**Table 1:** Questionnaire Response Rates by Province

Province	Number of Clinics in Province	Number of Clinics Responding	Response Rate (%)
British Columbia (BC)	4	3	75
Alberta (AB)	1	1	100
Saskatchewan (SK)	1	1	100
Manitoba (MB)	1	1	100
Ontario (ON)	14	12	86
Quebec (QC)	5	3	60
New Brunswick (NB)	1	1	100
Nova Scotia (NS)	1	1	100
Total	28	23	82

Of the responding 23 clinics, 18 (78%) were willing to accept HIV-positive individuals for consultation. When asked with reference to the couple, 18/23 (78%) clinics would accept couples in which the female partner was HIV-positive, 17/23 (74%) would accept couples in which the male partner was HIV-positive, and 17/23 (74%) would accept couples in which both partners were HIV-positive for consultation.

Although greater than 70% of clinics were willing to see HIV-positive individuals or couples in consultation, substantially less had actually seen any HIV-positive men (11/23, 48%) or women (10/23, 43%) in the previous 12 months. Of those who answered, 12 clinics stated that these individuals were seeking care for fertility problems and 7 stated that they were seeking strategies for risk reduction in achieving pregnancy (reducing the risk of sexual transmission to either partner while achieving pregnancy).

Regarding services offered, there was regional variation, with clinics in every province offering infertility investigations but only clinics located in five provinces offering fertility treatments if either or both partners were HIV-positive. A smaller number of clinics were willing to offer risk reduction techniques in achieving pregnancy. Table 2 presents the services available based on province. For couples in which the male partner was HIV-positive and the female partner HIV-negative, 17/23 (74%) clinics would offer investigations, 12/23 (52%) would offer fertility treatments, and 10/23 (43%) would offer risk reduction techniques. The numbers were very similar for couples in which the female partner was HIV-positive and the male partner HIV-negative, with 16/23 (70%) clinics offering investigations, 11/23 (48%) offering fertility treatments, and 10/23 (43%) offering risk reduction techniques. If both partners were HIV-positive, access decreased, with 15/23 (65%) clinics offering investigations, 8/23 (35%) offering fertility treatments, and 6/23 (26%) offering risk reduction techniques. Using logistic regression, we analyzed the likelihood of being offered investigations, treatments, or risk reduction techniques based on which partner was HIV-positive (Table 3). There were no statistically significant differences in these likelihoods regardless of whether the male partner, female partner, or both partners were HIV-positive (p > .05 for all comparisons).

**Table 2:** Services Available Provincially for HIV-Positive Individuals

Province (N = 23)	Infertility Investigations Offered	Infertility Treatments Offered	Risk Reduction Techniques Offered
BC (N = 3)	Yes	No	No
AB (N = 1)	Yes	Yes	Yes
SK (N = 1)	Yes	Yes	Yes
MB (N = 1)	Yes	Yes	No
ON (N = 12)	Yes	Yes	Yes
QC (N = 3)	Yes	No	No
NB (N = 1)	Yes	No	No
NS (N = 1)	Yes	Yes	Yes

**Table 3:** Differences in Investigation and Treatment Rates Based on HIV Status

	Male HIV+/Female HIV-	Male HIV-/Female HIV+	Male HIV+/Female HIV+	P value
Infertility investigations offered (%)	74	70	65	0.82
Infertility treatments offered (%)	52	48	35	0.47
Risk reduction techniques offered (%)	43	43	26	0.38

With respect to specific advanced reproductive technologies available, the most commonly available technique was intrauterine insemination (IUI) for couples in which the female partner was HIV-positive, with 12/23 (52%) providing this service (at least one clinic in seven out of eight provinces). Other procedures were less commonly available, with sperm washing for couples in which the male partner was HIV-positive offered in only 6/23 (26%) clinics in four provinces and in vitro fertilization (IVF) for couples in which the female partner was HIV-positive offered in only 4/23 (17%) clinics in two provinces. Eight clinics (35%) reported being able to offer any combination of technologies for HIV-infected couples. Finally, 12/23 (52%) clinics (at least one clinic in seven out of eight provinces) were willing to offer donor sperm to single HIV-positive women.

In addition to assessing clinic experience with and willingness to provide services for HIV-positive individuals and couples, there were also questions in the survey about clinic policies with respect to potentially infected specimens. Only 5/23 (22%) clinics in two provinces had written policies regarding the investigations of HIV-positive individuals, but 16/23 (70%) had policies governing treatment. A very small proportion of clinics used separate times of the day or week (3/23, 13%) or had separate facilities (2/23, 9%) to handle potentially contaminated specimens.

## Discussion

In this national survey of fertility clinics carried out across Canada, access to infertility investigations and treatments was limited for HIV-positive men and women, and was regionally dependent. While greater than 70 per cent of clinics reported being willing to see HIV-positive individuals, only approximately half had actually seen any in the previous 12 months. Roughly 50 per cent of clinics offered fertility treatments to this group of individuals, with the most commonly available being IUI. Access to sperm washing and IVF was much more limited.

As life expectancy among HIV-positive people has increased, fertility desires and pregnancy planning have emerged as important issues in this community. Arising from this is a need to facilitate access to advanced reproductive technologies and fertility treatments. Two broad groups of patients are likely to request these options. The first group consists of couples in which only one partner is infected with HIV (serodiscordant couples) who would like to have children but who wish to avoid the risk of transmission to the uninfected partner. These couples may or may not be subfertile. The second group consists of serodiscordant couples who are subfertile and have tried to conceive spontaneously without success, subfertile couples in which both partners are infected, same-sex couples, and single HIV-positive men and women wishing to have children.

Since the early years of the HIV epidemic, the treatment of fertility issues in couples in which one or both partners was HIV-positive has been controversial. Major issues included the possibility of mother-to-child transmission and the likelihood that the parent(s) might die before the child reached adulthood. Both of these issues have become less of a problem in recent years. HIV-positive individuals now have aggressive treatment options available to them which have resulted in dramatic declines in mortality. Furthermore, advances in the management of HIV in pregnancy have decreased rates of mother-to-child transmission to very low levels. In one of the first randomized placebo-controlled trials in pregnant women, the use of zidovudine monotherapy in pregnancy, during labor and delivery, and postpartum to the infant, resulted in a drop in transmission rates from 25.5% with no treatment to 8.3% in the treatment group [15]. Subsequent studies have shown that in the setting of cART and an undetectable viral load, transmission rates are as low as 1% to 2% or less [16-18].

With these favorable statistics, it has been argued that it is unethical to withhold fertility care and/or assisted reproductive technologies to couples in which one or both partners is HIV-positive [6-8]. There are many other serious and potentially fatal diseases affecting women in their reproductive years, and fertility treatments are rarely refused in these cases. Conditions such as longstanding insulin-dependent diabetes mellitus or lupus nephritis have been cited as examples in which the long-term health of the mother may be at higher risk than with HIV [7]. Similarly, there are conditions such as Tay-Sachs disease in which the risk of transfer to children is approximately the same as HIV infection even before interventions that lowered this rate. Couples with these risks are often still encouraged to consider pregnancy, and not denied access to assisted reproductive technologies. Denying HIV-positive individuals and couples the opportunities for fertility counseling and treatments may lead them to distance themselves from the medical community, and to engage in behavior that puts one or both partners at risk [19,20]. The results of our survey revealed that the majority of clinics in Canada (78%) were willing to see HIV-positive individuals in consultation, although only 48% had actually seen an HIV-positive man and 43% had actually seen an HIV-positive woman in the preceding 12 months. Some clinics in smaller communities reported that although they were willing to see HIV-positive men and women, they were not able to offer investigations or treatments, so they encouraged these individuals to be seen in more urban areas in adjacent provinces.

In a couple where the female partner is infected and the male partner is not, there are two commonly employed strategies used to achieve a pregnancy. The first is to have unprotected intercourse, which places the male partner at risk of seroconversion. The second is to use self-insemination techniques at home, which have variable success rates. Couples who fail to conceive in this way may require more formal assistance with options such as cycle monitoring to use timed insemination, IUI or IVF. Our results showed that IUI was the most commonly available technique in Canada, with 52% of responding clinics offering this service to couples in which the female partner was HIV-positive (at least one clinic in seven out of eight provinces).

In a couple where the male partner is infected and the female partner is not, she is at risk of acquiring HIV infection if they try to achieve pregnancy through unprotected intercourse, with an approximate risk of 0.1% to 0.2% per unprotected act [21]. In a series of 104 pregnancies achieved through natural conception in HIV-positive men with HIV-negative women partners, no seroconversions occurred within the first three months following conception, but two women seroconverted at seven months of pregnancy and two others converted postpartum [22]. This study was performed before viral load testing was available, so viral load status of these men is not known. However, it has been shown that blood and genital tract viral loads may not correlate well, so even a low blood viral load may not translate into low semen viral loads [23]. A safer alternative is the use of sperm washing techniques which separate actual spermatozoa from seminal fluid, which harbors the virus. Sperm itself does not express significant levels of HIV receptors, so it is unlikely to be a major target of HIV infection [23]. To date, 300 healthy children have been born after more than 3,000 cycles of sperm washing and IUI or IVF, with no reported seroconversions in either partner or children [9-12]. Most reported cycles are from Europe, with only a small number from the United States, and no published Canadian series. In our survey, access to sperm washing and IVF was very limited, with these technologies only available in a small number of clinics. Six clinics (26%) in four provinces offered sperm washing, and only four clinics (17%) in two provinces offered IVF.

In a couple where both partners are infected, avoidance of unprotected intercourse is still advised, to prevent potential transmission of more aggressive virus between partners. These couples also may benefit from fertility services.

In every province, there were more clinics willing to offer investigations than treatments to HIV-positive individuals. According to respondents, this does not reflect a lack of willingness to treat HIV-positive people, but reflects the technical difficulties with the handling of potentially infected specimens and training of staff. Although there is good evidence for the safety and efficacy of advanced reproductive technologies in HIV-infected couples, access to clinics providing these services in Canada is limited. The safest way to offer these services may be to separate HIV-infected patients in either time and/or space from HIV-negative individuals [18,19]. Some clinics have even built separate laboratories with separate equipment to achieve this goal [19]. A very small proportion of clinics in Canada have adopted these policies, with 3/23 (13%) using separate times of the day or week and 2/23 (9%) having separate facilities to handle potentially contaminated specimens. Survey respondents replied that this was an expensive and logistically difficult strategy to put into practice.

For all combinations of couples, more clinics offered fertility treatments than risk reduction techniques in achieving pregnancy. This finding is interesting, and may indicate a greater willingness among clinics to intervene in cases where there are fertility problems (for example, blocked fallopian tubes or low sperm counts) compared with couples for whom fertility is not a problem, but who are seeking safer methods to become pregnant. Future research should focus on this aspect of access to care for couples in which one or both partners is HIV-positive.

In both the United Kingdom and Australia, studies similar to this one have been performed to evaluate the accessibility of fertility services for HIV-infected couples [13,14]. Each of these were national surveys of all clinics in the country. In the United Kingdom, the study was performed in 2001 and 57/75 (76%) of clinics responded [13]. Only 39% of clinics had seen an HIV-positive individual in the previous year (48% and 43% for men and women, respectively, in our study). If the male partner was HIV-positive, 58% of clinics would offer investigations and 44% would offer treatment (74% and 52% in our study). If the female partner was HIV-positive, 44% would offer investigations and 25% would offer treatment (70% and 48% in our study). In Australia, the study was performed in 2002 and 31/52 (60%) of clinics responded [14]. Only 45% of clinics had seen an HIV-positive man or woman in the preceding 12 months. With respect to investigations, 28/31 clinics stated they were willing to offer investigations. If the male partner was HIV-positive 87% would offer treatment, and 77% would offer treatment if the female partner was HIV-positive. However, between one-quarter and one-third stated there were certain specific treatments that would not be offered. Further, there was no breakdown in either study as to specifically which treatments would or would not be offered. The findings of the current study differ from these studies in some important ways. First, our response rate is higher, at 82%. Second, our study was performed several years later, so perhaps attitudes have changed and access has improved over time. In our study, more clinics had seen an HIV-positive individual. More clinics were willing to offer investigations and treatments compared to the United Kingdom study, and less clinics stated that there were treatments that wouldn't be offered compared to the Australian study.

This study has several strengths. It is the first national survey of fertility clinics in Canada to assess access to investigations and treatments for HIV-positive men and women. The response rate of 23/28 (82%) was high, with only 5/28 clinics not responding. The surveys were completed by the Medical or Laboratory Directors of the clinics, and these individuals would be the most knowledgeable about clinic experience with certain populations.

There are some limitations to the current study. With surveys, there is the chance of recall bias, and we had no way of confirming the responses to our questionnaires. All clinics associated with the Canadian Fertility and Andrology Society were used for the study, but there may be other clinics in Canada that are not affiliated with this organization and therefore were missed. Finally, we used an unvalidated survey as no validated instrument existed, although we did model it on two previously published surveys and attempted to validate it ourselves prior to use.

## Conclusions

There is a paucity of data available on the types of services available for HIV-positive men and women in the arena of pregnancy planning and infertility. This is an important area for future investigation and research. The findings of this study are provocative, and demonstrate that access to fertility investigations and treatments for HIV-positive individuals is limited in Canada, and that this access is regionally dependent. Policy makers and health care professionals caring for these people should focus on developing strategies to increase access.

## Competing interests

The authors declare that they have no competing interests.

## Authors' contributions

All three authors (MY, HM, MR) contributed significantly to the conception, design, and performance of the study. MY sent out the questionnaires and collected the responses, and wrote the first draft of the manuscript. All authors participated in manuscript revision, and gave final approval for publication.
